# The Impact of Precaution and Practice on the Performance of a Risky Motor Task

**DOI:** 10.3390/bs3030316

**Published:** 2013-06-26

**Authors:** Hila Keren, Pascal Boyer, Joel Mort, David Eilam

**Affiliations:** 1 Department of Zoology, Tel Aviv University, Ramat-Aviv 69978, Israel; 2 Departments of Psychology and Anthropology, Washington University, St Louis, MO 63130, USA; E-Mail: pboyer@wustl.edu; 3 711th Human Performance Wing, US Air Force Research Laboratory, WPAFB, OH 45433, USA; E-Mail: joel.mort@wpafb.af.mil

**Keywords:** emotion, affect, anxiety, precaution, cognition, behavior

## Abstract

The association between threat perception and motor execution, mediated by evolved precaution systems, often results in ritual-like behavior, including many idiosyncratic acts that seem irrelevant to the task at hand. This study tested the hypothesis that threat-detection during performance of a risky motor task would result in idiosyncratic activity that is not necessary for task completion. We asked biology students to follow a particular set of instructions in mixing three solutions labeled “bio-hazardous” and then repeat this operation with “non-hazardous” substances (or *vice versa*). We observed a longer duration of the overall performance, a greater repertoire of acts, longer maximal act duration, and longer mean duration of acts in the “risky” task when it was performed before the “non-risky” task. Some, but not all, of these differences were eliminated when a “non-risky” task preceded the “risky” one. The increased performance of idiosyncratic unnecessary activity is in accordance with the working hypothesis of the present study: ritualized idiosyncratic activities are performed in response to a real or illusionary threat, as a means to alleviate anxiety.

## 1. Introduction

Threat evokes an emotional state that leads to significant changes in behavior, which in the face of present danger may be generalized into freezing, fleeing, or fighting (for review, see [1]). Humans and other animals, however, are also capable of perceiving potential threats and subsequently undertake precautionary measures in order to avoid them. The usual response to potential threat perception is anxiety, an affective state of apprehension, tension, distress, or uneasiness of mind about uncertain outcomes. Anxious humans and other animals may perform routine actions at a slower pace, invest greater cognitive resources (attention) in performance, and monitor more closely the effects of each act [2,3]. For example, vigilant scanning, which is alertness or readiness to detect events that could be of serious concern to an animal and its companions [4], has been described in a wide range of animals (e.g., ostriches: [5]; and antelopes: [6]). In humans, anxiety may result in a repeated and precise performance of acts, and this presumably generates a sense of controllability and predictability, with a consequent reduction in anxiety [7]. Threat perception may consequently shape the performance of motor sequences into a ritual-like behavior (see [8] and [2] for review). It has been suggested that anxiety and the consequent precautionary behavior, and specifically risk assessment, enable an individual to practice defensive means in physical safety, so that when a real threat occurs, the organism is primed for the appropriate defensive reaction [9]. In light of this notion, in the present study we examined how individual humans behave when notified that a certain task is hazardous, compared to individuals who are informed that the same task is non-hazardous.

The connection between threat-perception and intensified scrutiny of motor performance is a characteristic of obsessive-compulsive disorder (OCD)—a type of anxiety disorder [10,11] characterized by a motivation to act in a secure way in order to reduce perceived potential danger [12]. When OCD behavior is compared with non-OCD behavior, the commonness of a motor act can be used as a proxy for its relevance to the function of the task. That is, acts that are performed by both the OCD and the non-OCD individuals (“common acts”) are considered as compulsory for the completion of that task, and therefore as “functional”. In contrast, idiosyncratic acts that are performed only by the OCD or by the non-OCD individual are considered as “non-functional” or “non-pragmatic”, as reflected in the completion of the task without these acts by the other person [2,13,14,15,16,17]. Using the shared performance (commonness) of acts as a proxy for act functionality in the context of the current task, we then extended it from OCD to daily motor activities, revealing a bimodal distribution in act-sharing, which may represent a synthesis of functionality and individuality. Specifically, acts that were common to all the individuals performing the same task were the attributes that gave the task its label (“making coffee,” “lighting a cigarette,” *etc.*). Again, these acts were considered “functional” or “pragmatic” since no individual accomplished the task without them. Other acts were idiosyncratic, and since some individuals accomplished the task without them, they were not compulsory for task completion and accordingly considered as “non-functional” or “non-pragmatic” acts [18]. It should be noted that both the common and the idiosyncratic acts were performed within the spatio-temporal domain of the task. For example, when one basketball player (but not others) kisses the ball within the sequence of acts before making a free throw, then kissing the ball becomes an idiosyncratic act in this sport-related task. Theoretically, if five out of 10 basketball players would kiss the ball, this would give that act 50% commonness and 50% idiosyncrasy, while another act could have 60% idiosyncrasy, and so on, altogether establishing a continuous gradient of commonness-idiosyncrasy for the repertoire of acts that are relevant for accomplishing a specific motor task. The more common an act, the more it is necessary for the task, and therefore considered as more “functional” (or “pragmatic”). Nevertheless, in various motor tasks it has become apparent that there is no such gradient, and most of the act repertoire dichotomizes to either common (“functional”) acts that are performed by all actors, or idiosyncratic (“non-functional”) acts performed by only 1–2 actors. Only a minority of the non-functional acts are performed by several actors [18]. 

What may be important in determining the potential adaptive value of ritualized behavior is the predictable temporal placement of idiosyncratic acts in ritualized action sequences. Idiosyncratic “non-functional” acts are aggregated at the beginning of sport-related tasks such as basketball free-throws or weightlifting (e.g., kissing the basketball before taking a free throw); whereas in compulsive rituals of OCD patients the idiosyncratic non-functional acts are aggregated at the end of the motor task (e.g., repeatedly checking the door frame after locking the door). Together, idiosyncratic acts either precede or follow the pragmatic set of common (“functional”) acts, as if they are a transitional phase of warming-up and cooling-down for the core functional acts of the motor task [19]. These preparatory and confirmatory phases have also been found in routine daily motor tasks such as donning a buttoned shirt, lighting a cigarette, making coffee, *etc.* [18]. Similar transitional phases have been described in animal behavior, in which displacement activities irrelevant to the current task facilitated transitions between different behaviors [20]. In other words, their role is to function as a “cut-off” signal, diverting attention from the current behavior and decreasing the associated motivational arousal, as preparation for the performance of the subsequent task [21]. Altogether, idiosyncratic, seemingly unnecessary, and non-functional acts have been found in a large range of motor activities (daily motor routines, [18]; sports, [19]; cultural rituals, [22]; OCD patients, [2]). While the number and temporal aggregation of these acts at the beginning or end of motor tasks differ in predictable ways among the above domains of behavior, they seem to emerge, in accordance with the hypothesis of Boyer and Liénard, via an activated “precaution system” [8]. It was suggested that this system is geared to the detection of and reaction to inferred threats, and that it is distinct from fear-systems geared to respond to manifest danger. 

In the study presented here we sought to examine the association between threat perception and motor execution. Our working hypothesis rested on two principles: (i) activating precaution brain mechanisms results in ritualized behavior [8,12,23]; and (ii) ritualized behavior is characterized by idiosyncratic acts [13,19]. Accordingly, we hypothesized that non-clinical participants would accomplish specific sets of instructed actions, presented as potentially dangerous, differently to how they would accomplish a similar set of instructed actions presented as non-dangerous. Specifically, we expected that the participants would spend more time, perform more acts, and engage in more idiosyncratic acts, when handling ostensibly bio-hazardous substances, compared to handling non bio-hazardous ones.

## 2. Method

### 2.1. Participants

Twenty biology students (14 women, 6 men; age 24–40 years) were requested to participate in the study. The students were not dependent on the experimenters of this study, nor were they compensated for participating. The study was carried out at a laboratory in the I. Meier Segals Garden for Zoological Research at Tel Aviv University, Israel, and was approved by the Institutional Helsinki Committee for Human Experimentation at Tel Aviv University; all participants signed informed consent before the study. 

### 2.2. Design and Procedure

The 20 participants were randomly divided into two groups. Each participant performed, in a hood, two sequential simple laboratory tasks according to written instructions provided at the beginning of each task (see Appendix I). The task consisted in mixing specific volumes of unidentified solutions, labeled as A, B, C, and D, according to instructions. Ten participants were informed that the solutions were bio-hazardous. Upon completion of the task they were asked to perform the protocol again, but this time they were informed that the solutions were “non-hazardous”. Participants were provided with the usual protective means (gloves, lab gown, working in a hood) and were not aware that in both trials all the solutions were simply water with food coloring dyes. Another group of 10 participants underwent the same experiment but with trials in the reverse order (first no-hazard then hazard trials). Testing took about 20 minutes per participant, and was video-recorded throughout by the experimenter with a hand-held camera (Panasonic SDR-H20), until the participant had completed the two trials. 

### 2.3. Data Acquisition and Analysis

Video files were analyzed using the ethological coding and analysis *Observer* software (by Noldus Information Technologies, NL). Behavioral analyses were carried out first by listing the objects and the locations in the trial, and then scoring the acts performed with or at each object/location. For example, the solution tubes were the objects, and the acts performed on these objects could be “grab,” “pour,” *etc.* In this procedure, the objects/locations represent the targets for action (action space), and the acts are the elementary buildings blocks of the motor task that are performed with/at each of these target objects/locations. The lists of acts and objects/locations were arranged by an observer with years of experience in this form of parsing. Listing was validated by another experienced observer. After encoding the list of objects/locations and acts performed with/at these objects/locations into the *Observer*, behavior was manually scored by the observer during playback of the video files (usually in slow-motion mode). For each act, scoring included the name of the act, the object/location with/at which it was performed, and the time at which it had started and ended. From the encoded acts, the following parameters were extracted: 

*Trial duration:* Time from the beginning of the first act to the end of the last act in a trial.

*Common acts:* These were performed in both trials by the same individual. 

*Idiosyncratic acts:* These were performed by a specific individual in only one of her/his trials. 

*Total number of acts:* The total number of acts, both common and idiosyncratic, in a trial (repetitions included). 

*Act repertoire:* Some acts (common or idiosyncratic) were performed repeatedly. The set of acts performed in a trial, repetitions excluded, was termed repertoire. 

*Act duration:* The mean, maximum, and minimum duration of each act type (e.g., the duration of acts such as “grab a tube” or “pour into a tube”).

*Maximal act duration:* The duration (s) of the longest act in a trial has been previously found to best discriminate between compulsive and non-compulsive performance [13], following the notion that compulsive behavior is a product of over-activation of the precaution system [8], or of high-security motivation [12,23]. 

*Proportional number of idiosyncratic acts:* The ratio between the number of idiosyncratic acts and the total number of acts (idiosyncratic + common) was calculated for each individual.

*Proportional duration of idiosyncratic acts:* The time that each individual spent in performing idiosyncratic acts out of the total trial duration.

### 2.4. Statistical Analysis

A two-way ANOVA with repeated measures was used in most comparisons, with risk as a within-group factor (hazardous *vs.* non-hazardous trials) and order of trials (hazardous first and then non-hazardous, or *vice versa*) as a between-group factor. None of the tested parameters deviated significantly from normal distribution, as verified by a Kolmogorov–Smirnov test. Proportion data were transformed into arc-sinus of their square root. Statistical analysis was performed using STATISTICA 8 software, with the alpha level set to 0.05.

## 3. Results

**Figure 1:**
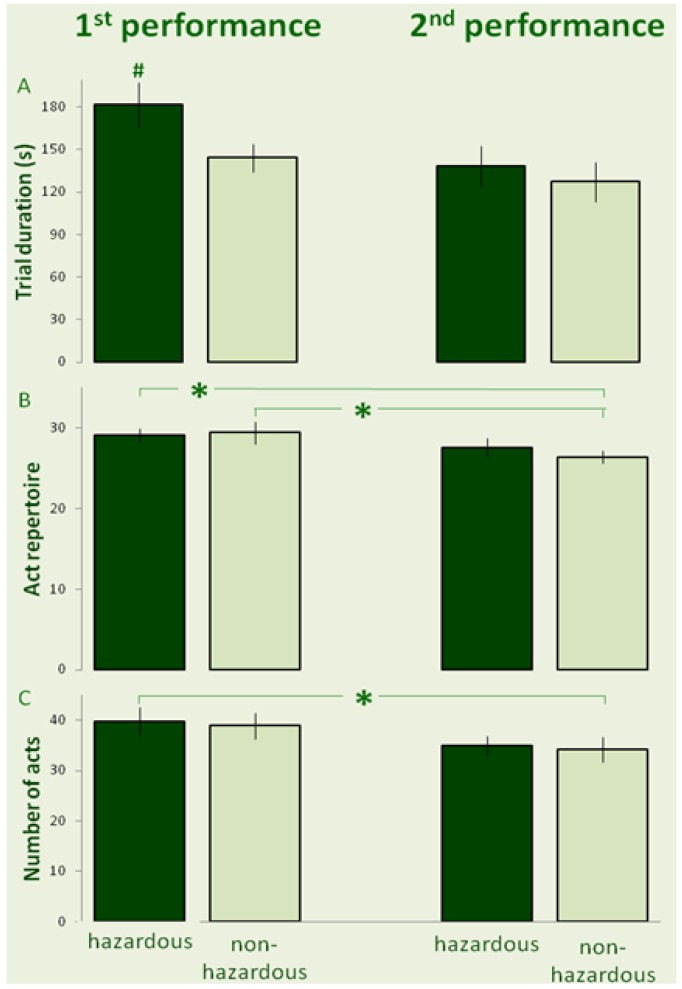
The mean (± SE) of trial duration (**A**), act repertoire (**B**), and the total number of acts (**C**) are depicted for hazardous (dark bars) and non-hazardous trials (light bars) during the first performance (left-hand insets) and second performance (right-hand insets). # indicates a significant difference compared to all other trials. (* indicates a difference between the trials, connected by the horizontal line.)

The mean (± SE) trial duration of each group is depicted in Figure 1A. Repeated measures ANOVA revealed a significant effect of risk (*F*_1,18_ = 6.59; *p* = 0.019; partial ƞ^2^ = 0.26), no significant effect of order of trials (*F*_1,18_ = 0.72; *p* = 0.411; partial ƞ^2^ = 0.04), and a significant interaction between risk and order of trials (*F*_1,18_ = 10.25; *p* = 0.005; partial ƞ^2^ = 0.36). A Fisher LSD test revealed that a “hazardous” trial that was performed first took a significantly longer duration than all other trials. Act repertoire (number of different acts excluding repetition) and the total number of acts (including repetitions) are depicted in Figure 1B, C, respectively. For act repertoire, repeated measures ANOVA revealed no effect of risk (*F*_1,18_ = 0.83; *p* = 0.374; partial ƞ^2^ = 0.04) and no effect of trial order (*F*_1,18_ = 0. 32, *p* = 0.578; partial ƞ^2^ = 0.02). However, there was a significant interaction between risk and trial order (*F*_1,18_ = 20.78; *p* < 0.001; partial ƞ^2^ = 0.54). A Fisher LSD test revealed that act repertoire was significantly greater in the first “hazardous” or “non-hazardous” trials than in the subsequent “non-hazardous” trial. For the total number of acts in a trial, repeated measures ANOVA revealed no effect of risk (*F*_1,18_ = 0.31; *p* = 0.587; partial ƞ^2^ = 0.02), and no effect of trial order (*F*_1,18_ = 0.01, *p* = 0. 987; partial ƞ^2^ < 0.01), but a significant interaction between risk and trial order (*F*_1,18_ = 9.54; *p* = 0.006; partial ƞ^2^ = 0.35). A Fisher LSD test revealed that when the “hazardous” trial was first, the total number of acts in that trial was significantly greater than in the subsequent “non-hazardous” trial (Figure 1C).

**Figure 2:**
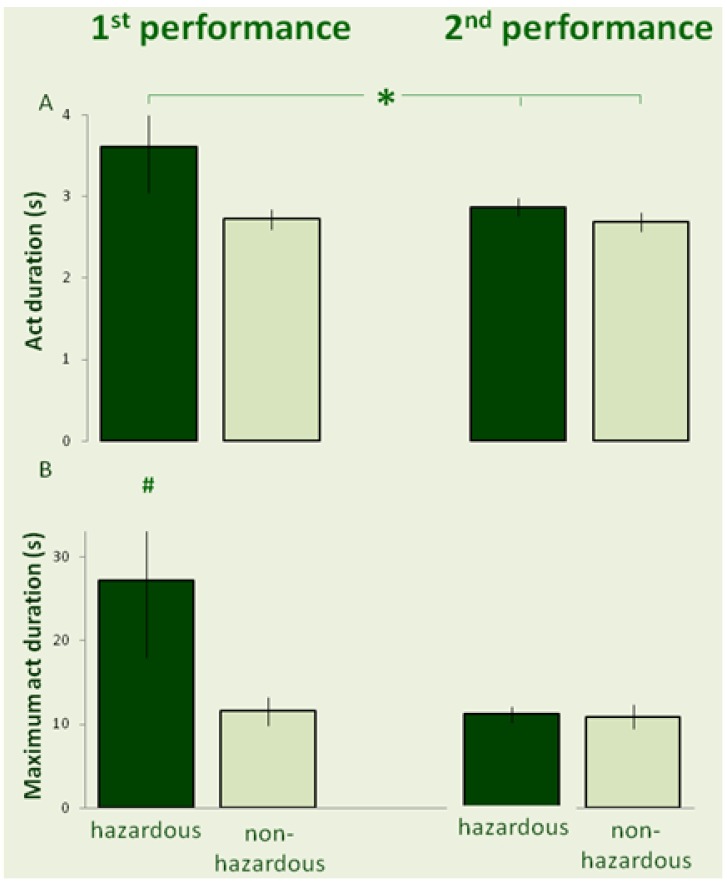
The mean (± SE) of act duration (A), and maximum act duration (B) are depicted for hazardous (dark bars) and non-hazardous trials (light bars) during the first performance (left-hand insets) and second performance (right-hand insets). # indicates a significant difference compared to all other trials. (* indicates a difference between the trials, connected by the horizontal line.)

The mean act duration is depicted in Figure 2A. A repeated measures ANOVA revealed no significant effect of trial order (*F*_1,18_ = 1.5; *p* = 0.234; partial ƞ^2^ = 0.08), no significant effect of risk (*F*_1,18_ = 2.11; *p* = 0.163; partial ƞ^2^ = 0.11), and a significant interaction (*F*_1,18_ = 4.92; *p* = 0.041; partial ƞ^2^ = 0.21). A Fisher LSD test revealed that the mean duration of acts was significantly longer in the first hazardous trial than in the second trial (either hazardous or non-hazardous), but not longer than the act duration in the first performance of a non-hazardous trial. 

The maximal act duration, for each trial for each individual, was also compared by a repeated measures ANOVA, since a previous study [16] had suggested that this is the best discriminator between compulsive and non-compulsive performance, following the notion that compulsive behavior is a product of sustained anxiety. Here we found no significant effect of order (*F*_1,18_ = 2.52; *p* = 0.129; partial ƞ^2^ = 0.12), a marginal effect of risk (*F*_1,18_ = 4.38; *p* = 0.051; partial ƞ^2^ = 0.19), and significant interaction between order and risk (*F*_1,18_ = 4.73; *p* = 0.043; partial ƞ^2^ = 0.21). Fisher LSD test revealed that the maximal act duration in “hazardous” first trials was significantly longer than in all other trials. Altogether, a first “hazardous” trial resulted in a longer mean duration of acts and longer maximum of act duration. 

The results shown in Figure 1, Figure 2 demonstrate that the effect of risk in the first performance of the task was manifested in longer duration of the overall performance, greater repertoire of acts, longer maximal act duration, and longer mean duration of acts. However, the performance of a similar “non-hazardous” task prior to the “hazardous” task abolished the impact of risk, and most of the measured parameters did not differ in individuals who performed first a “non-hazardous” motor task and then performed the same task with “hazardous” materials. 

**Figure 3:**
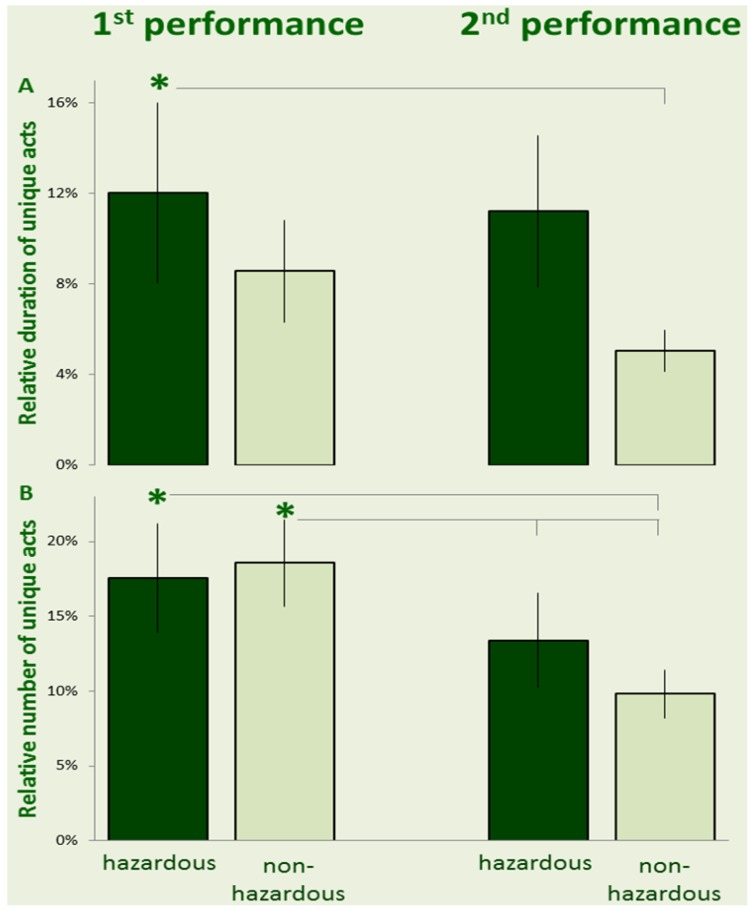
The mean (± SE) relative duration of idiosyncratic acts (**A**), and the relative number of idiosyncratic acts (**B**) are depicted for hazardous (dark bars) and non-hazardous trials (light bars) during the first performance (left-hand insets) and second performance (right-hand insets). (* indicates a difference between the trials, connected by the horizontal line.)

As explained in the Methods section, acts for each individual were classified into those performed in both trials (common acts) and those performed in only one trial (idiosyncratic acts). For each individual, we calculated the proportion of idiosyncratic acts out of her/his total number of acts, the proportion of time that the individual spent in performing idiosyncratic acts out of the total trial duration, and the proportion of idiosyncratic act repertoire out of the entire repertoire. These data were calculated as proportions in order to avoid a possible bias due to differences in total trial duration. For time spent in performing idiosyncratic acts, a repeated measures ANOVA revealed no significant effect of risk (F_1,18 _= 0.95; *p* = 0.344; ƞ^2^ = 0.05) or trial order (F_1,18 _= 0.26; *p* = 0.625; ƞ^2^ = 0.01), but a significant interaction (F_1,18 _= 6.1; *p* = 0.024; ƞ^2^ = 0.25). A Fisher LSD test revealed that the relative duration of idiosyncratic acts during the first performance of a “hazardous” trial was significantly longer than in the subsequent “non-hazardous” trial, whereas the relative duration of idiosyncratic acts was not significantly different when the “hazardous” trial was preceded by a “non-hazardous” trial (Figure 3A). For the number of idiosyncratic acts out of the total number of acts, a repeated measures ANOVA revealed no significant effect of risk (F_1,18 _= 0.08; *p* = 0.784; partial ƞ^2^ < 0.01), no significant effect of trial order (F_1,18 _= 0.28; *p* = 0.602; partial ƞ^2^ = 0.02), and a significant interaction (F_1,18 _= 16.54; *p* < 0.001; partial ƞ^2^ = 0.48). A Fisher LSD test revealed that both first trials (either “hazardous” or “non-hazardous”) included a greater number of idiosyncratic acts than their respective second trial (Figure 3B). It should be noted, that over 80% of trial duration and 80% of the total number of acts were devoted to common acts, regardless of hazard and regardless of order, as reflected in the low values seen in Figure 3A,B. 

## 4. Discussion

The present study tested the hypothesis that precaution during the performance of a perceived risky motor task is manifested in longer overall duration (slower pace), more acts, and specifically more idiosyncratic acts unnecessary for task completion. We examined the influence of two factors on task performance; (i) risk (hazardous or non-hazardous task) and (ii) order of tasks (which of the tasks is performed first: hazardous or non-hazardous). The “risky” task consisted in mixing three solutions labeled “bio-hazardous”, while in the non-risky task the same solutions were labeled “non-hazardous”. We found that a “risky” task that was performed first significantly differed in various parameters from a subsequent “non-risky” task, and from the performance of a risky task following a similar preceding, but non-risky, task. In the following discussion we scrutinize and distinguish between the impacts of risk and of practice on motor performance. 

### 4.1 The Impact of Practice on Performance

The present study revealed that a single initial performance of a task, regardless of its risk, results in improved subsequent performance, manifested as faster completion and fewer unnecessary acts. This could be merely motor learning, which is known to be primarily affected by practice [24]. Specifically, practice is generally considered the single most important factor in the acquisition of motor skills [25,26], leading to faster initiation and execution of movements and thereby improved performance. Likewise, the first performance of the task in the present study involved a greater repertoire of acts than the repeated performance, regardless of perceived danger. Each individual appears to have learned from experience that some acts were unnecessary and could be skipped; and such a learning process is typical of motor tasks [27]. Altogether, practicing a task improves performance, as if making the perceived task simpler, leading to a more automated performance, and requiring less attention [28,29]. Motor performance is limited, however, by factors that are not affected by practice, such as individual ability, mental capacity, and innate talent [30]. In pathologies such as Parkinson’s disease, this kind of limitation restricts rehabilitation to specific tasks only [31]. On the whole, the improved performance found here in the repeated task seems to be a typical product of practice, regardless of risk. It could also be argued, however, that this was confounded by the participants’ beliefs: specifically, one might argue that individuals who had performed the non-risky trial first would probably not believe that a quasi-identical manipulation in the second phase of the experiment was actually dangerous. Such an interpretation can only be ruled out by future studies disentangling these two factors. 

### 4.2 The Impact of Risk: Precaution is Manifested in Performing More Acts Than Necessary for Task Completion

The present results revealed that when the hazardous trial was performed first, it featured a longer duration, a greater repertoire of acts and total number of acts, longer duration of idiosyncratic unnecessary acts, and greater mean duration of the maximal act compared with the subsequent “non-hazardous” trial. These effects disappeared when the non-hazardous trials were performed first. This implies that the increase in the number of idiosyncratic acts that were unnecessary for task completion was the main manifestation of a sense of “risk”, but also depended of practice. Indeed, when the “hazardous” trial was performed first, both the sense of risk and the lack of practice accounted for the increase in other behavioral parameters. 

The longer duration of idiosyncratic unnecessary acts in “hazardous” trials is in accordance with the working hypothesis of the present study. As noted in the Introduction, such an increase is a salient feature of ritualized behavior, a product of activated brain precaution mechanisms [8,12,13,19,23,32,33,34]. Specifically, the notion that performing ritualized activities in response to real or illusionary threat as a means to alleviate anxiety has been introduced in several disciplines. Healthy humans engaging in ritualized and repeated behaviors have reported that these behaviors reduced their feelings of anxiety, fear, and discomfort, and increased their sense of control and security [35]. This is the core of conceptualizing ritualized and repeated behaviors as a coping strategy that alleviates anxiety in normal individuals as well as in those suffering from mental disorders [12,33,36] (see [2] for review). The problem with this hypothesis, however, is the lack of a precise measure of increased ritualization. In the present study we overcame this obstacle by using the commonality of acts as a proxy for their relevance to the task (see [2,13,18,19]). We considered an act as necessary for task completion when an individual performed it in both trials, and considered an act as idiosyncratic and unnecessary for task completion when that individual skipped this act in one of the trials, attesting that the task could be completed without the act. This classification of acts provided a means to demonstrate that tasks perceived as more risky indeed featured more acts that were not necessary for completion of that task. 

## 5. Conclusions

The results imply that a first performance of a task, be it hazardous or non-hazardous, includes more types of different acts (a greater repertoire) than in subsequent performances of the same task. It also includes more acts that seem irrelevant and unnecessary for the task (since they were skipped in one out of two repetitions of the task by the same participant). In addition, most of the duration parameters seemed also to be affected by the risk component as well as by the order of tasks, with the total task duration and the mean and maximum act duration being significantly longer in the first performed hazardous task than in all other trials. It seems that both the order of task performance and the risk component have an effect: task order has an effect on the number of different act types and on the number of irrelevant acts; while risk has an effect on task duration and on the mean and maximum duration of acts. It should be noted that in the present study, which was performed with ordinary healthy biology students who are familiar with such motor tasks, over 80% of trial duration and 80% of the total number of acts were devoted to acts that were relevant to the task, regardless of risk and practice history. This implies that the impact of both risk and practice on idiosyncrasy was marginal, and the main body of the task was pragmatic. It is this marginal component, however, that becomes exaggerated in other forms of ritualized behavior, where it can comprise the majority of the motor task, as evident in the high rate of idiosyncratic acts in cultural [22] and sport rituals [19], as well as in OCD [13,14,16,17]. 

## Appendix I—Trial Protocols

Participants were given the following written protocols and were requested to prepare a drug and control vehicle for one of our tests. Ten individuals were first given protocol 1 and then protocol 2; and another 10 individuals were first given protocol 2 and then protocol 1.

### Protocol 1—Preparing the Active Solution

Please read the directions and strictly follow the instructions. Make sure to wear gloves and lab coat and work in a hood.

#### Material and Tools:

50cc tube A—50cc tube B with bio-hazard solution (use with caution)—50cc tube C—Beaker—Measuring tube—Pipette

#### Directions:

1. Pour 10cc from tube A into the beaker.
2. Add 20cc from tube B into the beaker (**use with caution: solution in tube B is bio-hazardous**)
3. Shake carefully.
4. Pour 10cc from tube C into the beaker.
5. Shake and leave in the back of the hood.

Please use the lab sink to wash your hands at the end.

### Protocol 2—Preparing the Control Vehicle

Please read the directions and strictly follow the instructions. Make sure to wear gloves and lab coat and work in a hood.

#### Material and Tools:

50cc tube A—50cc tube B with bio-hazard solution (use with caution)—50cc tube C—Beaker—Measuring tube—Pipette

#### Directions:

1. Pour 10cc from tube A into the beaker.
2. Add 20cc from tube B into the beaker
3. Shake carefully.
4. Pour 10cc from tube C into the beaker.
5. Shake and leave in the back of the hood.

Please use the lab sink to wash your hands at the end.

Thank you for your help.
